# Relationship between temporal rhythm-based classification of atrial fibrillation and stroke: real-world vs. clinical trial

**DOI:** 10.1007/s11239-022-02638-0

**Published:** 2022-04-15

**Authors:** Wern Yew Ding, José Miguel Rivera-Caravaca, Francisco Marin, Vanessa Roldán, Gregory Y. H. Lip

**Affiliations:** 1 Liverpool Centre for Cardiovascular Science, University of Liverpool and Liverpool Heart & Chest Hospital, William Henry Duncan Building 6 West Derby Street, L7 8TX Liverpool, United Kingdom; 2 Department of Cardiology, Hospital Clínico Universitario Virgen de la Arrixaca, University of Murcia, Instituto Murciano de Investigación Biosanitaria (IMIB-Arrixaca), CIBERCV, Murcia, Spain; 3 Department of Hematology and Clinical Oncology, Hospital General Universitario Morales Meseguer, University of Murcia, Murcia, Spain; 4 Aalborg Thrombosis Research Unit, Department of Clinical Medicine, Aalborg University, Aalborg, Denmark

**Keywords:** Atrial fibrillation, Classification, AF type, Stroke, Risk, CHA_2_DS_2_-VASc, Real-world, Clinical trial

## Abstract

**Background:**

The risk of stroke according to clinical classification of atrial fibrillation (AF) remains poorly defined. Here, we assessed the impact of AF type on stroke risk in vitamin K antagonist-treated patients with AF in ‘real-world’ and ‘clinical trial’ cohorts.

**Methods:**

Post-hoc analysis of patient-level data from the Murcia AF Project and AMADEUS trial. Clinical classification of AF was based on contemporary recommendations from international guidelines. Study endpoint was the incidence rate of ischaemic stroke. Stroke risk was determined using CHA_2_DS_2_-VASc score and CARS. A modified CHA_2_DS_2_-VAS‘c’ score that applied one additional point for a ‘c’ criterion of continuous AF (i.e. non-paroxysmal AF) was calculated.

**Results:**

We included 5,917 patients: 1,361 (23.0%) real-world and 4,556 (77.0%) clinical trial. Baseline demographics were balanced in the real-world cohort but clinical trial participants with non-pAF (vs. pAF) were older, male-predominant and had more comorbidities. Crude stroke rates were comparable between the groups in real-world patients (IRR 0.72 [95% CI,0.37-1.28], *p* = 0.259) though clinical trial participants with non-pAF had a significantly higher crude rate of stroke events (IRR 4.66 [95%,CI,2.41-9.48], *p* < 0.001). Using multivariable analysis, AF type was not independently associated with stroke risk in the real-world (adjusted HR 1.41 [95% CI,0.80-2.50], *p* = 0.239) and clinical trial (adjusted HR 1.16 [95% CI,0.62-2.20], *p* = 0.646) cohorts, after accounting for other risk factors. There was no significant improvement in the CHA_2_DS_2_-VAS‘c’ compared to CHA_2_DS_2_-VASc score in either cohorts (*p* > 0.05).

**Conclusions:**

Overall, our results support the need for anticoagulation based on thromboembolic risk profile rather than AF type.

**Supplementary Information:**

The online version contains supplementary material available at 10.1007/s11239-022-02638-0.

## Introduction

The lifetime risk of atrial fibrillation (AF) is about 1 in 4 [1] and it has an estimated prevalence of between 2% and 4% in adults [2]. AF is now believed to be a marker of atrial cardiomyopathy ‘substrate’ that is associated with a prothrombotic state through various mechanisms [3]. The most widely accepted clinical classification of AF is according to temporal rhythm-based patterns, reflecting the notion that most patients initially suffer from transient episodes that prolong over time due to atrial substrate remodelling as the disease progresses; more recently the 4 S-AF scheme has been proposed to characterise AF, and incorporated into guidelines [3, 4].

Other markers of atrial cardiomyopathy such as left atrial dimension and fibrosis have been found to be independently associated with stroke risk [5–7]. Therefore, it may be speculated that patients with extended episodes of ‘continuous’ AF (persistent, long-standing persistent and permanent AF) may be at higher risk of stroke complications compared to paroxysmal AF (pAF) due to the degree of underlying cardiomyopathy and also the direct prothrombotic effects of AF *per se* [8]. Nonetheless, this remains an ill-defined area with previous studies having obtained conflicting results [9–12].

In this study, we assessed the impact of AF type on stroke risk in anticoagulated patients with AF in ‘real-world’ (Murcia AF Project) and ‘clinical trial’ cohorts (AMADEUS [Evaluating the Use of SR34006 Compared to Warfarin or Acenocoumarol in Patients With Atrial Fibrillation] trial).

## Methods

We analysed patient-level data from the Murcia AF Project and AMADEUS trial. The design of these studies have formerly been described [13, 14]. Briefly, the Murcia AF Project was an observational study that enrolled consecutive outpatients with AF who were on stable vitamin K antagonist (VKA) therapy (i.e. International Normalised Ratio [INR] of 2.0 to 3.0) during the preceding six months between May and December 2007 in a single-tertiary centre from the Southeast of Spain. The initial period of stable INR was intended to minimise heterogeneity, thus avoiding confounding factors due to differences in the quality of anticoagulation control at study entry. The AMADEUS trial was a multicentre, randomised, open-label non-inferiority study with blinded adjudication of outcomes comparing fixed-dose idraparinux and dose-adjusted VKA in patients with AF [15]. Recruitment occurred between September 2003 and July 2005.

Patients in each cohort were categorised into two groups based on ECG recordings at enrolment using the contemporary temporal rhythm-based classification system [4]. Paroxysmal AF (pAF) was defined as AF that terminates spontaneously or with intervention within seven days of onset. Non-paroxysmal AF (non-pAF) was defined as AF that lasted longer than seven days, including persistent, long-standing persistent and permanent AF subtypes. A complete medical history was documented at inclusion and the recorded parameters were used to determine stroke risk with CHA_2_DS_2_-VASc score [16] and CARS [17], and bleeding risk with HAS-BLED score [18]. A modified CHA_2_DS_2_-VAS‘c’ score that applied one additional point a ‘c’ criterion of continuous AF (ie. non-paroxysmal AF) was calculated for each individual patient.

The study endpoint was the incidence rate of ischaemic stroke during follow-up. In the Murcia AF Project, ischaemic stroke was defined as an abrupt onset of focal neurological deficit in a location consistent with the territory of a major cerebral artery due to an obstruction verified by imaging, surgery or autopsy. Major bleeding was defined according to the 2005 International Society on Thrombosis and Haemostasis (ISTH) [19]. All events in the AMADEUS trial were adjudicated by a central committee, who were blinded to original treatment assignment.

### Statistical analyses

Continuous baseline variables were expressed as median and interquartile range (IQR), and tested for differences with Mann-Whitney U test. Categorical variables were expressed as absolute frequencies and percentages, and tested for differences using chi-squared test. Crude event rates per 100-patient years (PYs) with their Poisson 95% confidence intervals (CIs) were calculated for stroke (and major bleeding) and a comparison between AF types was performed using incidence rate ratio (IRR). This was further analysed according to subgroups of patients based on the CHA_2_DS_2_-VASc score.

Multivariable Cox proportional hazards model was used to determine the effects of AF type on stroke risk after accounting for potential confounders with the CHA_2_DS_2_-VASc score, a well-recognised method of risk stratification in AF, and anticoagulation agent for patients in the AMADEUS trial. The predictive performance of the CHA_2_DS_2_-VAS‘c’ score for stroke events was investigated using receiver-operating characteristic curves, and tested against the CHA_2_DS_2_-VASc score. C-index was used to reflect the ability of scores to predict events. A two-sided *p* value of less than 0.05 was considered statistically significant. Analyses were performed using SPSS software version 24.0 (SPSS Inc., Chicago, Illinois, United States) and MedCalc v. 16.4.3 (MedCalc Software bvba, Ostend, Belgium).

## Results

**Fig. 1:**
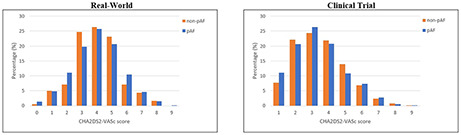
Distribution of CHA_2_DS_2_-VASc score among non-pAF vs. pAF in Real-World and Clinical Trial

We included 5,917 patients with AF: 1,361 (23.0%) real-world and 4,556 (77.0%) clinical trial. Real-world patients had a median age of 76 (IQR 71–81) years with 51.3% females compared to a median age of 71 (IQR 64–77) years with 33.5% females among clinical trial participants. Baseline demographics according to AF type for both cohorts are summarised in Table 1. Across both studies, CARS was similar in both groups (*p* > 0.05). The distribution of CHA_2_DS_2_-VASc score among patients with non-pAF vs. pAF is shown in Fig. 1. For clinical trial patients on VKA therapy, the median time-in-therapeutic range was 59 (IQR 45–71) in patients with non-pAF and 57 (IQR 44–80) in pAF.

**Table 1:** Baseline characteristics according to AF type in Real-World and Clinical Trial

Baseline characteristics	Real-World			Clinical Trial		
	**non-pAF (n = 182)**	**pAF (n = 1179)**	***p*** **value**	**non-pAF (n = 2922)**	**pAF (n = 1634)**	***p*** **value**
Age (years), median (IQR)	76 (70–81)	76 (71–81)	0.351	72 (65–77)	70 (63–76)	< 0.001
Female sex, n (%)	104 (54.1)	594 (50.4)	0.089	889 (30.4)	639 (39.1)	< 0.001
BMI (kgs/m^2^), median (IQR)	29.5 (26.1–32.9)	29.3 (26.5–32.8)	0.755	28.1 (25.3–31.4)	27.8 (25.2–31.3)	0.134
eGFR (ml/min/1.73 m^2^), median (IQR)	69.9 (55.3–84.4)	71.2 (59.0–86.0)	0.576	86.3 (69.7–94.2)	85.7 (67.7–93.6)	0.036
Comorbidities, n (%)						
Anaemia	36 (19.8)	218 (18.5)	0.678	150 (10.3)	96 (11.9)	0.240
Coronary artery disease	39 (21.4)	216 (18.3)	0.317	914 (31.3)	489 (29.9)	0.343
Diabetes mellitus	46 (25.3)	317 (26.9)	0.647	589 (20.2)	303 (18.5)	0.188
Heart failure	48 (26.4)	381 (32.3)	0.108	817 (28.0)	252 (15.4)	< 0.001
Hypertension	149 (81.9)	967 (82.0)	0.961	2200 (75.3)	1313 (80.4)	< 0.001
Previous ischaemic stroke or TIA	41 (22.5)	230 (19.5)	0.342	602 (20.6)	363 (22.2)	0.201
Risk profile, median (IQR)						
CHA_2_DS_2_-VASc score	4 (3–5)	5 (4–6)	0.961	3 (2–4)	3 (2–4)	0.009
CARS (%)	3.7 (2.5–5.7)	3.8 (2.7–5.6)	0.980	2.9 (2.0–5.0)	2.8 (1.9–8.1)	0.252
HAS-BLED score	2 (2–3)	2 (2–3)	0.488	1 (1–2)	2 (1–2)	0.112

AF, atrial fibrillation; BMI, body mass index; CKD, chronic kidney disease; eGFR, estimated glomerular filtration rate; IQR, interquartile range; pAF, paroxysmal AF; TIA, transient ischaemic attack

### Stroke and major bleeding rates

During a median follow-up period of 79.1 (IQR 52.2–96.1) months, there were a total of 130 (9.6%) ischaemic stroke and 250 (18.4%) major bleeding events in the real-world. The ischaemic stroke rates in patients with non-pAF and pAF were 1.10 (95% CI 0.59–1.88) per 100PYs and 1.53 (95% CI 1.26–1.83) per 100PYs, respectively (Table 2). There was no significant difference in the crude ischaemic stroke rates between the groups with an IRR of 0.72 (95% CI 0.37–1.28), *p* = 0.259. This finding was consistent across the various subgroups of patients based on CHA_2_DS_2_-VASc score (**eTable 1**). Similarly, major bleeding rates were comparable in both groups with an IRR of 0.95 (95% CI 0.63–1.38), *p* = 0.791.

**Table 2:** **Stroke and bleeding rates in Real-World and Clinical Trial cohorts**

Stroke and bleeding rates	non-pAF			pAF					
	**n**	**Event rate/100PYs**	**95% CI**	**n**	**Event rate/100PYs**	**95% CI**	**Incidence rate ratio**	**95% CI**	***p*** **value**
*Real-World*									
Stroke	13	1.10	0.59–1.88	117	1.53	1.26–1.83	0.72	0.37–1.28	0.259
Major bleeding	32	2.71	1.85–3.82	218	2.84	2.48–3.25	0.95	0.63–1.38	0.791
*Clinical Trial*									
Stroke	31	2.29	1.55–3.24	14	0.49	0.27–0.82	4.66	2.41–9.48	< 0.001
Major bleeding	79	5.86	4.64–7.31	34	1.20	0.83–1.67	4.89	3.23–7.55	< 0.001

CI, confidence interval; pAF, paroxysmal AF; PYs, patient-years

Over a median follow-up of 347 (IQR 186–457) days, there were a total of 45 (0.99%) ischaemic stroke and 113 (2.48%) major bleeding events in the clinical trial cohort (Table 2). The ischaemic stroke rates in patients with non-pAF and pAF were 2.29 (95% CI 1.55–3.24) per 100PYs and 0.49 (95% CI 0.27–0.82) per 100PYs, respectively. Participants with non-pAF had a significantly higher crude rate of ischaemic stroke events with an IRR of 4.66 (95% CI 2.41–9.48), *p* < 0.001. This finding was driven by an excess of ischaemic stroke rates in those with a CHA_2_DS_2_-VASc score of 4 to 5 (**e**Table 2). There was also elevated rates of major bleeds among these participants with an IRR of 4.89 (95% CI 3.23–7.55), *p* < 0.001.

### Multivariable analyses

On multivariable analyses, AF type had no significant impact on the ischaemic stroke risk in both real-world (adjusted hazard ratio 1.41 [95% CI 0.80–2.50], *p* = 0.239) and clinical trial (adjusted hazard ratio 1.16 [95% CI 0.62–2.20], *p* = 0.646) cohorts, after accounting for known risk factors using the CHA_2_DS_2_-VASc score and anticoagulation agent for clinical trial patients (Table 3).

**Table 3:** **Multivariable analyses of risk factors for stroke in Real-World and Clinical Trial**

Variables	Hazard ratio	95% CI	*p* value
*Real-World*			
CHA_2_DS_2_-VASc score	1.40	1.26–1.56	< 0.001
Non-paroxysmal AF	1.41	0.80–2.50	0.239
*Clinical Trial*			
CHA_2_DS_2_-VASc score	1.49	1.25–1.78	< 0.001
Non-paroxysmal AF	1.16	0.62–2.20	0.646
VKA vs. idraparinux	1.33	0.73–2.42	0.349

AF, atrial fibrillation; CI, confidence interval; VKA, vitamin K antagonist

### Predictive performance of CHA_2_DS_2_-VAS‘c’ vs. CHA_2_DS_2_-VASc

**Fig. 2:**
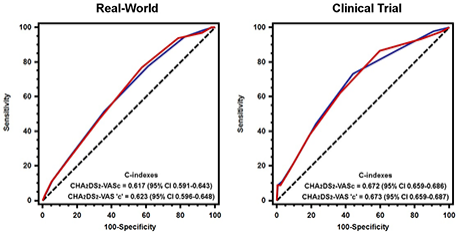
Receiver operating characteristic curves of CHA_2_DS_2_-VASc vs.CHA_2_DS_2_-VAS’c’ scores in Real-World and Clinical Trial Blue line = CHA_2_DS_2_-VASc; Red line = CHA_2_DS_2_-VAS‘c’. C-index comparison: Real-World (p = 0.312); Clinical Trial (p = 0.954)

The predictive performance of the CHA_2_DS_2_-VAS‘c’ score was modest in both the real-world (c-index 0.623 [95% CI 0.596–0.648]) and clinical trial (c-index 0.673 [95% CI 0.659–0.687]) (Fig. 2). There was no significant improvement in the CHA_2_DS_2_-VAS‘c’ compared to CHA_2_DS_2_-VASc score in either cohort (*p* > 0.05).

## Discussion

In this study of anticoagulated patients with AF, we found that patients with non-pAF had elevated ischaemic stroke rates in the clinical trial but not in the real-world setting. Second, rates in the former patient cohort was attributable to the presence of underlying risk factors rather than the presence of non-pAF itself, as reinforced by the higher crude risk of major bleeding. Third, inclusion of ‘continuous/chronicity of AF’ into the CHA_2_DS_2_-VASc score resulted in a minimal and non-significant improvement in the predictive performance of this model in both cohorts.

The findings presented here are consistent with other real-world studies and clinical trials. A sub-study of 6,706 patients in the ACTIVE W trial that compared oral anticoagulation (OAC) to combined antiplatelet therapy in AF showed no significant difference in the annual risk of thromboembolic events based on AF type [20]. Nevertheless, the authors only accounted for risk factors within the conventional CHADS_2_ score, thereby increasing the likelihood of residual confounders for stroke. Data from the EURObservational Research Programme-Atrial Fibrillation (EORP-AF) General Pilot Registry that compared 1-year outcomes among AF patients found that non-pAF was not independently associated with all-cause mortality, stroke or transient ischaemic attack, or major bleeding after a comprehensive adjustment for other risk factors [21]. The results were corroborated in a retrospective observational study of 7,156 patients with AF from the Loire Valley Atrial Fibrillation Project [10], though both studies were predominantly comprised of a cohort of European patients which may limit the generalisability of the reported outcomes. In this regard, a study of Asian patients in the Chinese Atrial Fibrillation Registry (CAFR) also demonstrated that AF type was not independently associated with thromboembolic risk [22].

In contrast to the aforementioned studies, Cho et al. reported that among 8,883 real-world patients with AF, those with non-pAF were older, male-predominant and had a higher prevalence of comorbidities which ultimately led to an increase in anticoagulation prescription and rhythm control treatment compared to those with pAF [9]. After accounting for these and other factors, the risk of ischaemic stroke remained 2-fold higher in patients with non-pAF compared to pAF over a follow-up period of 17 months. Nonetheless, the results may have been influenced by a low uptake of OAC, despite a high proportion of patients with at least a CHA_2_DS_2_-VASc score of 2, and the relatively low incidence of stroke in general. A pooled analysis of participants from the ACTIVE-A and AVERROES trials found that the pattern of AF was a strong independent predictor of stroke risk [12]. However, for inclusion into both these trials, participants had to be deemed unsuitable for VKA (or OAC) and there were no participants with a CHA_2_DS_2_-VASc score of 0 (or 1 in females), thus restricting clinical applicability of the results. An earlier meta-analysis of 99,996 patients from 12 studies reported that non-pAF was associated with a 1.4-fold and 1.5-fold increase in thromboembolism and mortality, respectively, even after multivariable adjustment with no significant difference in the rates of major bleeding [23].

Although the current clinical classification of AF is practical and widely adopted, some of the conflicting results in relation to stroke risk may be attributable to the inherent limitations of this system. For example, it has a poor ability to discriminate between AF patients on the basis of stage and severity of the underlying disease [24]. Furthermore, with the infrequent utilisation of continuous cardiac monitoring in most prior studies, the findings were likely subject to misclassification bias as the diagnosis of AF type was primarily reliant on the presence of patient-reported symptoms and snapshots of electrocardiographic tracings performed at widely spaced intervals [25]. Also, AF (or atrial tachyarrhythmia) burden, as detected using long-term implantable cardiac devices, may be a more reliable marker of atrial cardiomyopathy and thereby stroke risk. Nevertheless, this approach may not be feasible for the vast majority of patients who do not otherwise have an indication for these devices. Hence, the proposal of the 4 S-AF scheme in the new ESC guidelines to overcome some of these limitations [3, 4].

### Limitations

The findings from this study were based on a post-hoc analysis of the AMADEUS trial and a Caucasian population from a single-centre in the Murcia AF Project. Therefore, it should be interpreted with caution. Furthermore, in both studies, there was limited use of long-term continuous cardiac monitoring to determine AF type. Although the AMADEUS trial was performed in a historical cohort, the mainstay treatment for stroke prevention remains anticoagulation therapy. No other forms of treatment have been consistently shown to reduce stroke risk in the general AF cohort. Our findings may not be applicable to patients treated with direct oral anticoagulants and need further validation in a non-anticoagulated population. In general, the AMADEUS study population had a lower risk of stroke compared to other clinical trials, resulting in low event rates which may have contributed to a ‘false negative’ finding due to lack of statistical power. There was also the possibility of unmeasured confounding variables.

## Conclusions

Overall, our results support the need for anticoagulation based on thromboembolic risk profile rather than AF type. We did not find an association between the temporal rhythm-based patterns of AF and stroke risk in anticoagulated patients, suggesting that this should not be a consideration when assessing the need for anticoagulation in AF and that the presence of pAF (over non-pAF) should not offer false reassurance to clinicians. Rather, the focus should remain on identifying low-risk patients using proven stroke risk factors within the CHA_2_DS_2_-VASc score.

## Electronic Supplementary Material

Below is the link to the electronic supplementary material.

Supplementary Material 1

## Data Availability

The data that support the findings of this study are available from the corresponding author, GYHL, upon reasonable request.
