# Supplementary material for: Relationship between temporal rhythm-based classification of atrial fibrillation and stroke: real-world vs. clinical trial
Source: J Thromb Thrombolysis. 2022 Apr 15;54(1):1–6. doi: 10.1007/s11239-022-02638-0 (PMC9259516; doi:10.1007/s11239-022-02638-0)
Supplement: Supplementary file 1 — Supplementary Material 1 [file 11239_2022_2638_MOESM1_ESM.docx]

Supplemental Materials

Relationship between temporal rhythm-based classification of atrial fibrillation and stroke: real-world vs clinical trial

Short title: Stroke risk based on AF type

Wern Yew Ding^a*^ MRCP

José Miguel Rivera-Caravaca^a,b*^ RN, PhD

Francisco Marin^b^ MD, PhD

Vanessa Roldán^c#^ MD, PhD

Gregory Y. H. Lip^a,d#^ MD

[*joint first authors; ^#^ joint senior authors]

^a^ Liverpool Centre for Cardiovascular Science, University of Liverpool and Liverpool Heart & Chest Hospital, Liverpool, United Kingdom; ^b^ Department of Cardiology, Hospital Clínico Universitario Virgen de la Arrixaca, University of Murcia, Instituto Murciano de Investigación Biosanitaria (IMIB-Arrixaca), CIBERCV, Murcia, Spain; ^c^ Department of Hematology and Clinical Oncology, Hospital General Universitario Morales Meseguer, University of Murcia, Murcia, Spain; ^d^ Aalborg Thrombosis Research Unit, Department of Clinical Medicine, Aalborg University, Aalborg, Denmark.

Corresponding author:

Prof Gregory Y H Lip [gregory.lip@liverpool.ac.uk](mailto:gregory.lip@liverpool.ac.uk)

Full mailing address University of Liverpool

William Henry Duncan Building

6 West Derby Street

Liverpool L7 8TX, United Kingdom

Telephone number 0151 794 9020

**eTable 1. Effects of AF type on stroke rate stratified by CHA_2_DS_2_-VASc score in Real-World**

|  | **non-pAF** | | | **pAF** | | | **Incidence rate ratio** | **95% CI** | ***p* value** |
| --- | --- | --- | --- | --- | --- | --- | --- | --- | --- |
|  | **n** | **Event rate/100PYs** | **95% CI** | **n** | **Event rate/100PYs** | **95% CI** |  |  |  |
| CHA_2_DS_2_-VASc score 0 | 0 | 0 | 0 - 56.75 | 0 | 0 | 0 - 2.94 | NA | | |
| CHA_2_DS_2_-VASc score 1 | 0 | 0 | 0 - 51.96 | 2 | 0.45 | 0.06 - 1.64 | NA | | |
| CHA_2_DS_2_-VASc score 2 | 2 | 2.44 | 0.30 - 8.80 | 3 | 3.08 | 0.64 - 9.00 | 0.79 | 0.07 - 6.90 | 0.791 |
| CHA_2_DS_2_-VASc score 3 | 1 | 0.30 | 0.01 - 1.69 | 21 | 1.32 | 0.82 - 2.02 | 0.23 | 0.01 - 1.43 | 0.117 |
| CHA_2_DS_2_-VASc score 4 | 1 | 0.32 | 0.01 - 1.78 | 33 | 1.69 | 1.16 - 2.37 | 0.19 | 0.00 - 1.13 | 0.066 |
| CHA_2_DS_2_-VASc score 5 | 6 | 2.18 | 0.80 - 4.75 | 29 | 1.86 | 1.25 - 2.67 | 1.17 | 0.40 - 2.87 | 0.723 |
| CHA_2_DS_2_-VASc score 6 | 2 | 2.73 | 0.33 - 9.87 | 15 | 2.19 | 1.23 - 3.62 | 1.25 | 0.14 - 5.36 | 0.769 |
| CHA_2_DS_2_-VASc score 7 | 1 | 2.45 | 0.06 - 13.63 | 10 | 3.46 | 1.66 - 6.37 | 0.71 | 0.02 - 4.97 | 0.706 |
| CHA_2_DS_2_-VASc score 8 | 0 | 0 | 0 - 36.20 | 4 | 10.14 | 2.76 - 25.97 | NA | | |
| CHA_2_DS_2_-VASc score 9 | NA | | | 0 | 0 | 0 - 2.94 | NA | | |

AF, atrial fibrillation; CI, confidence interval; NA, not applicable; pAF, paroxysmal AF; PYs, patient-years.

**eTable 2. Effects of AF type on stroke rate stratified by CHA_2_DS_2_-VASc score in Clinical Trial**

|  | **non-pAF** | | | **pAF** | | | **Incidence rate ratio** | **95% CI** | ***p* value** |
| --- | --- | --- | --- | --- | --- | --- | --- | --- | --- |
|  | **n** | **Event rate/100PYs** | **95% CI** | **n** | **Event rate/100PYs** | **95% CI** |  |  |  |
| CHA_2_DS_2_-VASc score 1 | 0 | 0 | 0 - 2.63 | 1 | 0.49 | 0.01 - 2.71 | NA | | |
| CHA_2_DS_2_-VASc score 2 | 3 | 1.16 | 0.24 - 3.39 | 2 | 0.32 | 0.04 - 1.14 | 3.66 | 0.42 - 43.86 | 0.127 |
| CHA_2_DS_2_-VASc score 3 | 6 | 1.72 | 0.63 - 3.75 | 0 | 0 | 0 - 0.52 | NA | | |
| CHA_2_DS_2_-VASc score 4 | 8 | 2.68 | 1.15 - 5.27 | 5 | 0.82 | 0.27 - 1.90 | 3.28 | 0.95 - 12.74 | 0.027 |
| CHA_2_DS_2_-VASc score 5 | 7 | 4.18 | 1.68 - 8.61 | 3 | 0.75 | 0.16 - 2.20 | 5.56 | 1.27 - 33.31 | 0.005 |
| CHA_2_DS_2_-VASc score 6 | 3 | 2.77 | 0.57 - 8.10 | 2 | 1.25 | 0.15 - 4.51 | 2.22 | 0.25 - 26.59 | 0.370 |
| CHA_2_DS_2_-VASc score 7 | 0 | 0 | 0 - 8.35 | 1 | 1.57 | 0.04 - 8.75 | NA | | |
| CHA_2_DS_2_-VASc score 8 | 4 | 63.59 | 17.33 - 162.82 | 0 | 0 | 0 - 34.09 | NA | | |
| CHA_2_DS_2_-VASc score 9 | 0 | 0 | 0 - 409.88 | 0 | 0 | 0 - 203.81 | NA | | |

AF, atrial fibrillation; CI, confidence interval; NA, not applicable; pAF, paroxysmal AF; PYs, patient-years.
